# Ultrasmall PtMn nanoparticles as sensitive manganese release modulator for specificity cancer theranostics

**DOI:** 10.1186/s12951-023-02172-y

**Published:** 2023-11-18

**Authors:** Guoqiang Guan, Huiyi Liu, Juntao Xu, Qingpeng Zhang, Zhe Dong, Lingling Lei, Cheng Zhang, Renye Yue, Hongchang Gao, Guosheng Song, Xian Shen

**Affiliations:** 1 Department of Gastrointestinal Surgery, Key Laboratory of Diagnosis and Treatment of Severe Hepato-Pancreatic Diseases of Zhejiang Province, The First Affiliated Hospital of Wenzhou Medical University, Oujiang Laboratory, Wenzhou, 325000 Zhejiang China; 2 State Key Laboratory for Chemo/Bio-Sensing and Chemometrics, College of Chemistry and Chemical Engineering, Hunan University, Changsha, 410082 China

**Keywords:** Ultrasmall alloy nanoparticle, Ultrasensitive, H_2_O_2_-free oxidase enzyme-like activity, High field magnetic resonance imaging, Ferroptosis

## Abstract

Manganese-based nanomaterials (Mn-nanomaterials) hold immense potential in cancer diagnosis and therapies. However, most Mn-nanomaterials are limited by the low sensitivity and low efficiency toward mild weak acidity (pH 6.4–6.8) of the tumor microenvironment, resulting in unsatisfactory therapeutic effect and poor magnetic resonance imaging (MRI) performance. This study introduces pH-ultrasensitive PtMn nanoparticles as a novel platform for enhanced ferroptosis-based cancer theranostics. The PtMn nanoparticles were synthesized with different diameters from 5.3 to 2.7 nm with size-dominant catalytic activity and magnetic relaxation, and modified with an acidity-responsive polymer to create pH-sensitive agents. Importantly, R-PtMn-1 (3 nm core) presents “turn-on” oxidase-like activity, affording a significant enhancement ratio (pH 6.0/pH 7.4) in catalytic activity (6.7 folds), compared with R-PtMn-2 (4.2 nm core, 3.7 folds) or R-PtMn-3 (5.3 nm core, 2.1 folds), respectively. Moreover, R-PtMn-1 exhibits dual-mode contrast in high-field MRI. R-PtMn-1 possesses a good enhancement ratio (pH 6.4/pH 7.4) that is 3 or 3.2 folds for T_1_- or T_2_-MRI, respectively, which is higher than that of R-PtMn-2 (1.4 or 1.5 folds) or R-PtMn-3 (1.1 or 1.2 folds). Moreover, their pH-ultrasensitivity enabled activation specifically within the tumor microenvironment, avoiding off-target toxicity in normal tissues during delivery. In vitro studies demonstrated elevated intracellular reactive oxygen species production, lipid peroxidation, mitochondrial membrane potential changes, malondialdehyde content, and glutathione depletion, leading to enhanced ferroptosis in cancer cells. Meanwhile, normal cells remained unaffected by the nanoparticles. Overall, the pH-ultrasensitive PtMn nanoparticles offer a promising strategy for accurate cancer diagnosis and ferroptosis-based therapy.

**Graphical Abstract:**

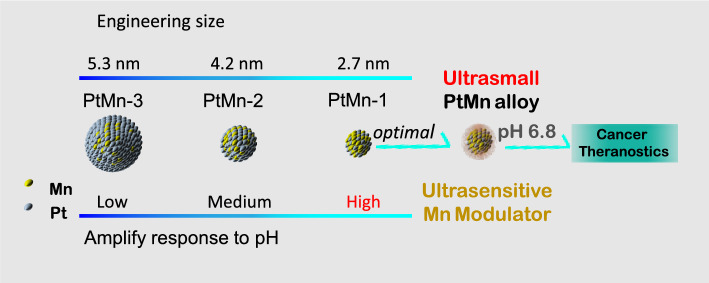

**Supplementary Information:**

The online version contains supplementary material available at 10.1186/s12951-023-02172-y.

## Introduction

Manganese-based nanomaterials (Mn-nanomaterials) offer a versatile platform for a wide range of nanomedicine applications in cancer diagnosis and therapies [1–13]. On one hand, Mn-nanomaterials such as MnO, MnO_2_, PtFe@MnO, and others have been developed as magnetic resonance imaging (MRI) contrast agents for biomedical imaging [1–6]. They possess five unpaired electrons, strong paramagnetic performance, long electronic relaxation time, and labile water exchange of Mn^2+^ ions, enabling applications in tumor diagnosis, Ca^2+^ activity detection, and monitoring tumor progression [1–3, 14, 15]. On the other hand, manganese (Mn), an essential micronutrient, exists in multiple oxidation states (+ 2, + 3, + 4) and participates in electron transfer reactions, molecular oxygen activation, storage, transport, and other important biological processes [1, 14]. Mn-nanomaterials exhibit intrinsic activities similar to peroxidase (POD), glutathione peroxidase (GPx), catalase (CAT), and superoxide dismutase (SOD) due to their variable Mn states and morphology [16–18]. Through Mn-mediated Fenton reactions, efficient glutathione (GSH) depletion, and oxygen (O_2_) generation, Mn-nanomaterials can be utilized in various therapies including photodynamic therapy, photothermal therapy, magnetic hyperthermia therapy, chemodynamic therapy, sonodynamic therapy, radiotherapy, chemotherapy, gene therapy, starvation therapy, ferroptosis, immunotherapy, and combination therapies [12, 19–35]. However, their “non-selective” catalytic effect in producing reactive oxygen species (ROS) often leads to significant “off-target” toxicity in normal tissues during delivery processes.

Due to the acidic nature of the tumor microenvironment (pH 6.4–6.8) compared to healthy tissue (pH 7.4), several acidity-activated ferroptosis agents have been developed [36–43]. However, most pH-triggered ferroptosis agents are insensitive to the relatively weak acidity found in tumors, resulting in inefficient catalytic activity enhancement ratios (pH 6.4/pH 7.4) [18, 43, 44]. The typical Fenton/Fenton-like reaction requires sufficient acidity (pH 3.0–6.0) and abundant hydrogen peroxide (H_2_O_2_) to ensure a high catalytic reaction rate [18, 43, 45]. However, the mildly acidic conditions (pH 6.4–6.8) resulting from upregulated glycolytic metabolism during tumorigenesis are not conducive to triggering Fenton/Fenton-like reactions [46–49]. Consequently, the therapeutic effects relying on these inadequate reactions are greatly limited [50–52]. Furthermore, the intratumoral concentration of H_2_O_2_ (commonly 50–100 μM) is insufficient to sustain the continuous generation of reactive oxygen species, leading to inadequate lipid peroxidation. Therefore, there is a pressing need to develop pH-ultrasensitive activatable ferroptosis strategies (pH 6.4–6.8) that do not rely on H_2_O_2_.

**Scheme 1:**
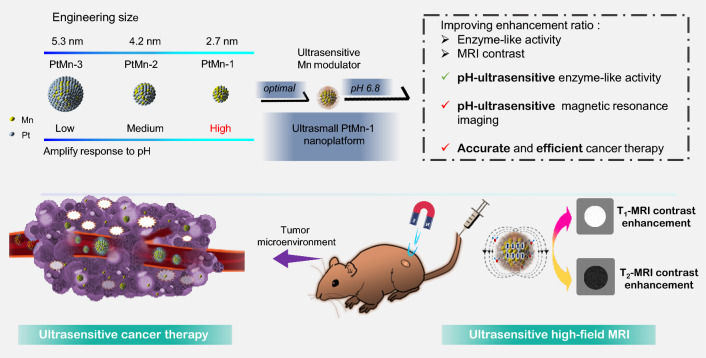
Schematic illustration of engineering size synthesis of uniform small PtMn nanoalloy from 5.3 to 2.7 nm for high enhancement ratio of oxidase-like activity and magnetic resonance imaging contrast. Ultrasmall PtMn-1 nanoplatform could be used as a pH-ultrasensitive ferroptosis agent for high-field magnetic resonance imaging and efficient cancer therapy

To address these issues, we have engineered a series of uniform-sized PtMn nanoparticles (PtMn-1, PtMn-2, PtMn-3) with different diameters (2.7 ± 0.6, 4.2 ± 0.7, and 5.3 ± 0.6 nm, respectively) by adjusting the volume ratio of 1-octadecene/dibenzyl ether (Scheme 1). We further modified these PtMn nanoparticles with an acidity-responsive polymer to create pH-sensitive agents (R-PtMn-1, R-PtMn-2, and R-PtMn-3). Interestingly, we found that R-PtMn-1 exhibited a higher enhancement ratio (pH 6.0/pH 7.4) of oxidase-like catalytic activity (6.7 folds), compared with those of larger-sized nanoparticles (R-PtMn-2, 3.7 folds or R-PtMn-3, 2.1 folds). The high enhancement ratio allows for efficient production of ROS and lipid peroxidation, leading to improved therapeutic effects in cancer therapy. Furthermore, R-PtMn-1 also demonstrated a noticeable enhancement contrast ratio (pH 6.4/pH 7.4) of threefold in T_1_-magnetic resonance imaging (MRI) and 3.2-fold in T_2_-MRI. In comparison, the enhancement contrast ratios in T_1_-MRI for R-PtMn-2 and R-PtMn-3 were 1.4-fold and 1.1-fold, respectively, while in T_2_-MRI, they were 1.5-fold and 1.2-fold respectively. The high enhancement contrast ratios in MRI imaging contribute to a higher signal-to-noise ratio, highlighting the relevant biological phenomena. Therefore, ultrasmall R-PtMn-1 nanoparticles (< 3 nm core) demonstrate pH-ultrasensitive MRI imaging and catalysis enhancement without requiring H_2_O_2_.

Remarkably, the pH-ultrasensitive oxidase-like catalysis of R-PtMn-1 is activated specifically within the tumor microenvironment (extracellular pH 6.4–6.8). Additionally, R-PtMn-1 exhibits pH-ultrasensitive enhanced T_1_-/T_2_-high field (7 T) MRI signals in tumors, which are sufficiently sensitive for effective signal activation. In contrast, R-PtMn-1 demonstrates no detectable oxidase activity and weak imaging signals in normal tissue, thereby reducing off-target toxicity and false signals in healthy tissue. Our development of pH-ultrasensitive high-field MRI-integrated therapeutic agents allows for accurate imaging and ferroptosis-based therapy through T_1_-/T_2_-MRI signal changes.

## Materials and methods

### Synthesis of PtMn nanoparticles

Typically, Pt(acac)_2_ (40 mg) was mixed into the solution of 1-octadecene (ODE) and dibenzyl ether (DE) (12 mL) in a 100 mL oblique three-neck ball flask. The transparent mixture solution was stirred intensely (over 300 rpm/min) and kept at 90 °C for over 1 h under the high-purity argon (99.99%). Then quickly added Mn(acac)_2_ (22.5 mg), using an amount of OA and OLA of 0.7 mmol. Next, the solution was further heated to about 205 ℃ and kept for over 0.75 h. The black solution was refluxed at 300 °C for over 2 h, then naturally cooled to room temperature, and washed excess ethanol and acetone in equal amounts 3 times by high-speed centrifugation (11,000 rpm/min, 20 min). After centrifugation, the nanoparticles were dispersed in 3 mL tetrahydrofuran for further characterization. The different-sized PtMn nanoparticles (PtMn-1, PtMn-2, PtMn-3) were prepared following the same procedure but using a different volume ratio of ODE and DE (1:0, 1:1, 0:1), respectively.

### Synthesis of polymers

PEG-RAFT, the pH-responsive polymer or non-pH-responsive polymer was prepared according to the previous report [53, 54]. The pH-responsive polymer was prepared using a reversible addition–fragmentation chain transfer (RAFT) polymerization strategy. PEG-RAFT (30 mg), DPA (154 mg), and AIBN (0.15 mg) were dissolved in 3 mL of dioxane and added into a flask. The flask was sealed under dry argon and was kept at 70 °C for over two days. After the reaction, the solution was dialyzed (MWCO: 3.5 KDa) using ultrapure water. Finally, the solution was lyophilized to obtain a pH-responsive polymer.
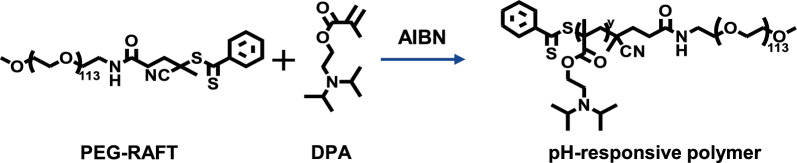


For the synthesis of non-pH-responsive polymer, MM (72 mg) which was replaced with DPA added into dioxane in the flask. The other procedures were similar to those for preparing pH-responsive polymer.
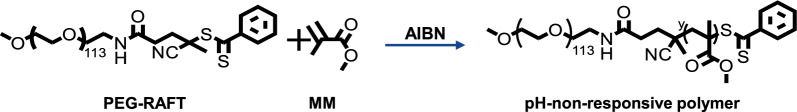


### Samples for MALDI-TOF and GPC characterization

2 mg pH-responsive polymer was dispersed in 1 mL tetrahydrofuran for Matrix Assisted Laser Desorption Ionization Time of Flight Mass Spectrometry (MALDI-TOF). 20 mg pH-responsive polymer was used for Gel Permeation Chromatography (GPC). The mobile phase used in the relative molecular weight test was tetrahydrofuran.

### Preparation of polymer-coated PtMn

For preparing pH-responsive polymer-coated PtMn, 50 μg of different-sized PtMn nanoparticles (PtMn-1, PtMn-2, PtMn-3) and 2 mg of pH-responsive polymer were dissolved into 1 mL of tetrahydrofuran (THF). The solution was sonicated for about 20 min and was quickly added into 4 mL of ultrapure water, followed by another 20 min of sonication. Subsequently, tetrahydrofuran was evaporated, resulting in a polymer-coated PtMn solution (R-PtMn-1, R-PtMn-2, or R-PtMn-3). The resulting solution was washed with water over 3 times using a centrifugal filter tube.

For the preparation of non-pH-responsive polymer-coated PtMn-1 nanoparticles, the main procedure was similar to that for preparing responsive polymer-coated PtMn nanoparticles, except using 2 mg of non- pH-responsive polymer.

### Samples for XPS characterization

0.1 mL tetrahydrofuran of PtMn-1 (2 mg/mL) was added in HEPES buffer (10 ×, 5.4) at room temperature for 1 h. After centrifugation, the nanoparticles were dispersed in 0.1 mL tetrahydrofuran. The solution was dropped on the silicon wafer and dried at room temperature for further XPS characterization.

### Measurement of metal ions released from PtMn

R-PtMn-1 or Nr-PtMn-1 (0.1 mL, Mn: 0.75 mg/mL), was incubated in 900 μL of HEPES (4-hydroxyethylpiperazine ethanesulfonic acid buffer saline) with various buffer (2 ×, pH = 4.4, 5.4, 6.4, 7.4) at 37 °C for different time points, respectively. Then the mixture solution was filtered by a centrifugal filter tube (3 KDa) and the aliquot of the filtrate was collected to determine the concentration of Pt and Mn via inductively coupled plasma–mass spectrometry (ICP-MS).

### Measurement of DLS and zeta potential

R-PtMn-1 (Mn: 5 μg/mL) was incubated in 0.1 mL of HEPES buffer with various buffers (2 ×, pH = 4.4, 5.4, 6.4, 7.4) for 12 h. R-PtMn-1, R-PtMn-2, or R-PtMn-3 (Mn: 0.1 μg/mL) dispersed in H_2_O, DPBS, HEPES, or DMEM for 12, 24, and 48 h, respectively. Then, the above solution was diluted (1/40) for measuring DLS. 0.1 mL of Nr-PtMn-1 or R-PtMn-1 (Mn: 5 μg/mL) was incubated in 0.1 mL of HEPES buffer with various buffers (2 ×, pH = 4.4, 5.4, 6.4, 7.4) for 6 h. Then, the above solution was diluted (1/40) for measuring zeta potential.

### Measurement of OXD activity in solution

To test catalytic activity via 3,3ʹ,5,5ʹ-tetramethylbenzidine (TMB) assay, 50 μL of Nr-PtMn-1, R-PtMn-1, R-PtMn-2 or R-PtMn-3 (50 μg/mL) was incubated in 50 μL of TMB (1.5 mM) and 50 μL of various buffer (10 ×, pH = 5.4, 6.0, 6.4, 6.8, 7.4,) for 1 h. For ox-TMB of R-PtMn-1 in solution, the absorption of 100 μL of the mixture which was incubated at various time points was recorded at 650 nm with an ultraviolet–visible (UV–Vis) spectrometer to reveal the catalytic activity.

To test the dynamic process of catalytic activity, 50 μL of R-PtMn-1, R-PtMn-2, or R-PtMn-3 (50 μg/mL) was incubated in 50 μL of TMB (1.5 mM) and 50 μL of HEPES buffer (10x, pH = 6.0). After incubation for different times (0–30 min), the absorption of TMB_ox_ was recorded at 650 nm by UV–Vis spectrometer.

### Measurement of GSH consumed

Various HEPES buffer (0.2 mL, 1x, pH = 7.4, 6.4, 5.4, or 4.4) containing Nr-PtMn-1 or R-PtMn-1 (50 μg/mL) and GSH (2 mM) was prepared and incubated at 37 ℃ for 12 h, respectively, followed by centrifugation. Then, 50 µL of supernatant was collected and further incubated with 50 µL of colorimetric 5,5′-dithiobis-2-(nitrobenzoicacid) (DTNB) [~ 1.2 mg/mL in dimethyl sulfoxide (DMSO)] for about 15 min. The above solution was diluted (1/6) for measurement of absorption at 412 nm (OD) to determine GSH consumed content via the following Eq. 1:

1 $$GSH\, comsumed\, content \left(\%\right)=\frac{O{D}_{control}-O{D}_{sample}}{O{D}_{control}}\times 100 \%$$

### Measurement of T_1_/T_2_-relaxation time

Various HEPES buffers (0.2 mL, 1 ×, pH = 7.4, 6.4, 5.4, 4.4) containing R-PtMn-1, R-PtMn-2 or R-PtMn-3 (Mn: 50 μg/mL) was incubated at 37 ℃ for 1 h. Various HEPES buffers (0.2 mL, 5 ×, pH = 7.4, 6.8, 6.4, 5.4, 4.4) containing R-PtMn-1 (Mn: 25, 50, 100, 200 μg/mL) was incubated at 37 ℃ for 6 h. Then, T_1_ or T_2_-relaxation time was tested via Bruker Minispec analyzer (60 MHz, Bruker, Germany).

For T_1_- or T_2_-MRI phantom imaging, various HEPES buffers (0.2 mL, 5 ×, pH = 7.4, 6.8, 6.4, 5.4, 4.4) containing R-PtMn-1 (Mn: 12.5, 25, 50, 100 μg/mL) was incubated at 37 ℃ for 6 h. Then, those samples were scanned by using a Bruker 7 T-MRI scanner with using T_1_-MRI sequence (field of view = 30 mm × 30 mm, size = 256 × 256, slice thickness = 0.7 mm, repetition time (T_R_) = 225.58 ms, and effective echo time (T_E_) = 4.5 ms) or T_2_-MRI sequence (field of view = 30 mm × 30 mm, size = 256 × 256, slice thickness = 0.7 mm, T_R_ = 2500 ms, T_E_ = 35 ms).

### Cellular experiment

The mouse breast carcinoma (4T1) cells, mouse colorectal cancer (CT26) cells as cancer cells, and human embryonic kidney cell line (HEK293 cells) as normal cells were incubated on the cell culture plate in Dulbecco’s Modified Eagle Medium (DMEM) containing 1% penicillin/streptomycin and 10% of fetal bovine serum at 37 °C with 5% CO_2_.

### Intracellular ROS evaluation

To assess the intracellular ROS production, 4T1 cells pre-seeded in optical cultured dishes were treated with Nr-PtMn-1 or R-PtMn-1 (30 μg/mL) pre-seeded in optical cultured dishes for 6 h. 4T1 cells pre-seeded in optical cultured dishes were treated with R-PtMn-1 (30 μg/mL) pre-seeded in optical cultured dishes for 2, 4, or 6 h. 4T1 cells pre-seeded in optical cultured dishes were treated R-PtMn-1 (0, 7.5, 15, 30 μg/mL) pre-seeded in optical cultured dishes for 4 h. HEK293 cells pre-seeded in optical cultured dishes were treated with R-PtMn-1 (30 μg/mL) pre-seeded in optical cultured dishes for 6 h. After being washed with DPBS over 3 times, those cells were stained with DCFH-DA (10 μM) and Hoechst (1 μg/mL) for 0.5 h, respectively. Then, the fluorescent emission of DCFH-DA (E_x_ = 488 nm, Em = 530 nm) was observed using a confocal laser scanning microscope (CLSM) to test intracellular ROS. The relative fluorescence intensity was measured by ImageJ software.

### Intracellular LPO evaluation

To assess the intracellular LPO production, 4T1 cells pre-seeded in optical cultured dishes were treated with Nr-PtMn-1 or R-PtMn-1 (30 μg/mL) pre-seeded in optical cultured dishes for 2 h. 4T1 cells pre-seeded in optical cultured dishes were treated R-PtMn-1 (30 μg/mL) pre-seeded in optical cultured dishes for 0.5, 1, 1.5, or 2 h. 4T1 cells pre-seeded in optical cultured dishes were treated R-PtMn-1 (0, 7.5, 15, 30 μg/mL) pre-seeded in optical cultured dishes for 2 h. HEK293 cells pre-seeded in optical cultured dishes were treated with R-PtMn-1 (30 μg/mL) pre-seeded in optical cultured dishes for 2 h. After being washed with DPBS over 3 times, those cells were stained with liperfluo (10 μM) and Hoechst (1 μg/mL) for 0.5 h, respectively. Then, the fluorescent emission of liperfluo (Ex = 488 nm, Em = 500–550 nm) was detected using CLSM. The relative fluorescence intensity was measured by ImageJ software.

### Mitochondrial membrane potential evaluation

To evaluate the change of mitochondrial membrane potential, 4T1 cells were pre-seeded in optical cultured dishes were treated with Nr-PtMn-1 or R-PtMn-1 (40 μg/mL) for 2 h. After being washed with DPBS 3 times, those cells were stained with JC-1 (10 μM) for 0.5 h. The fluorescent emission of JC-1 (Ex = 514 nm, Em = 529 nm; Ex = 585 nm, Em = 590 nm) was detected using CLSM. The relative fluorescence intensity of JC-1 was measured by ImageJ software.

### Intracellular GSH evaluation

To test intracellular GSH content, 4T1 cells were pre-incubated in 6-well plates incubated with Nr-PtMn-1 or R-PtMn-1 (30 μg/mL) for 24 h. 4T1 cells pre-incubated in 6-well plates incubated with R-PtMn-1 (0, 7.5, 15, 30 μg/mL) for 24 h. 4T1 cells pre-incubated in 6-well plates incubated with R-PtMn-1 (30 μg/mL) for 6, 12, or 24 h. 4T1 cells were disrupted, and the cell lysate was frozen by liquid nitrogen and dissolved at 37 ℃ for three times. 40 μL of the supernatant was collected by centrifugation at 4 ℃ (10,000 rpm, 8 min) and was incubated with 280 μL of reagent 2 and 80 μL of reagent 3 from the GSH assay kit (BC1175, Solarbio) for 10 min. The absorption of the mixture was detected via a microplate reader at 412 nm (OD) and the concentration of GSH was calculated using the following Eq. 2:

2 $$GSH\, content \left(\%\right)=\frac{OD(sample)}{OD(control)}\times 100 \%$$

### Intracellular MDA evaluation

To measure intracellular malondialdehyde via malondialdehyde (MDA) assay kit, 4T1 cancer cells pre-seeded in a 6-well plate were incubated with Nr-PtMn-1 or R-PtMn-1 (30 μg/mL) for 24 h, respectively. Next, those cells were lysed and the cell lysate was collected. 50 μL of the supernatant was collected by centrifugation at 4 ℃ (12,000 rpm, 8 min) and then was mixed with 150 μL of working solution and 50 μL of reagent 3 from MDA assay kit (BC0025, Solarbio). The mixture was boiled for 1 h at over 95 ℃ and cooled to room temperature. 180 μL of supernatant was collected through centrifugation (10,000 rpm, 5 min), and the absorption of supernatant was measured via a microplate reader at 450, 532, and 600 nm (OD450, OD532, OD600). Subsequently, the concentration of MDA content was calculated according to Eqs. 3 and 4.

3 $$\Delta OD=OD\left(Test\right)-OD(Blank)$$

4 $$MDA =0.01\times (12.9\times \left(\Delta OD532-\Delta OD600\right)-2.58\times \Delta OD450)$$

### Intracellular WB assays

To measure GPX4, BID, or ACSL4 expression by western blot assay, 4T1 cancer cells pre-seeded in a 6-well plate were treated with Nr-PtMn-1 or R-PtMn-1 (30 μg/mL) for about 6 h, respectively. Afterward, those cells were washed with ice-cold DPBS, harvested and the cell lysate was boiled for over 10 min. Then the proteins were further transferred to a 0.45 μm polyvinylidene difluoride (PVDF) membrane. The PVDF membrane was blocked in Tris-buffered saline containing Tween 20 and 5% dry skim milk (TBST), and incubated with rabbit GPX4, BID, or ACSL4 antibody (1: 1000, Absin) and β-actin antibody (1: 1000, Servicebio) for 12 h at 4 ℃. The membrane was washed with TBST and followed by the secondary antibody (1: 1000, YiShan Biotech) incubation for over 1 h. Finally, the membrane was washed with TBST and the band of each protein was captured with an enhanced chemiluminescent detection system. We have quantified the GPX4, ACSL-4, BID, and β-actin levels by image j for calculating the relative GPX4, ACSL-4, and BID levels.

### Intracellular cytotoxicity evaluation

To investigate the inhibition of cellular viability, CT26 cells and 4T1 cells received the following treatment:

CT26 cells pre-seeded into 96-well plates were treated with R-PtMn-1, R-PtMn-2, or R-PtMn-3with various concentrations (0, 30, 60, 120, 240 μg/mL) for 24 h, respectively. 4T1 cells pre-seeded in a 96-well plate were treated with R-PtMn-1 (240 μg/mL) for different incubation times (0, 4, 10, 18, 30 h), respectively. 4T1 cells pre-seeded in a 96-well plate were treated with Nr-PtMn-1, R-PtMn-1, R-PtMn-2 or R-PtMn-3 at different concentrations (0, 30, 60, 120, 240 μg/mL) for 24 h, respectively.

HEK cells pre-seeded into 96-well plates were treated with R-PtMn-1 with various concentrations (0, 30, 60, 120, 240 μg/mL) for 24 h, respectively.

After being washed with DPBS 3 times, those cells were incubated with 200 μL of DMEM containing MTT (0.5 mg/mL) for 3 h. After that, the solution was removed and each well was added with 180 μL of DMSO. After incubation at 37 ℃ for over 0.5 h, the absorption at 490 nm was detected via a microplate reader, and the relative cell viability was calculated according to the standard MTT method.

### Cancer imaging in vivo

All animal experiments were approved by the Institutional Animal Care and Use Committee of Hunan University (SYXK 2018-0006).

For preparing the tumor model, female BALB/c mice were subcutaneously injected using about 50 μL DPBS solution with 4T1 or CT26 tumor cells (~ 1 × 10^6^).

For in vivo T_1_ or T_2_ MRI imaging, 4T1 tumor-bearing mice were *i.t.* injected with Nr-PtMn-1 or R-PtMn-1 (25 μL, Mn: 20 μg/mL) or *i.v.* injected with R-PtMn-1 (200 μL, Mn: 700 μg/mL), respectively. Then, those mice were immediately anesthetized with isoflurane in oxygen and scanned by 7 T-MRI scanners (PharmaScan 70/16 US, Burker), using T_1_-MRI sequence (size = 384 × 384, FOV = 30 mm × 30 mm, slice thickness = 0.7 mm, T_R_ = 230.5 ms, and T_E_ = 4.5 ms) or T_2_-MRI sequence (size = 256 × 256, FOV = 30 mm × 30 mm, slice thickness = 0.7 mm, T_R_ = 2500 ms, and T_E_ = 35 ms).

### Catalytic cancer therapy in vivo

For cancer therapy in vivo, both *i.t.* and *i.v. the* administration was performed. CT26 tumor-bearing female mice were randomly divided into 4 groups (n = 5) and followed the following administration: (1) None treatment as the control group; (2) Nr-PtMn-1 (25 µL, Mn: 0.7 mg/mL), (3) R-PtMn-1 (25 µL, Mn: 0.7 mg/mL, *i.t.*), (4) R-PtMn-1 (200 µL, Mn: 0.7 mg/mL, *i.v.*). 4T1 tumor bearing female mice were randomly divided into 2 groups (n = 5) and received the following administration: (1) None treatment as the control group; (2) R-PtMn-1 (200 µL, Mn: 0.7 mg/mL, *i.v.*). The body weights and tumor volumes of mice from each group were recorded every other day during the 14 days of study. The volume of the tumor was calculated as Length × Width^2^/2. All groups of mice were sacrificed on the 14th day, and then the tumor weight of each group was recorded. Representative tumors were taken out on the second day, and the main five organs of representative mice from each group were collected after the 14th-day post-injection for H&E and TUNEL staining, via the standard protocol, and examined using a Pannoramic MIDI microscope (3DHIESTECH, Hungary).

For DCFH-DA or liperfluo staining, those tissues were collected from mice for cryo-sections. Then, tumors’ slices were stained with DCFH-DA (10 µM, Ex = 488 nm, Em = 530 nm) and DPAI (1 μg/mL, Ex = 358 nm, Em = 461 nm) for about 2 h, respectively. Finally, the fluorescent confocal images of those tissues were collected by CLSM. The relative fluorescence intensity was measured by ImageJ software.

### Statistical analysis

Statistics analysis was shown as mean ± standard deviation (SD). All experiments were carried out at least three times. A significant difference (*p < 0.05, **p < 0.01, ***p < 0.001) was done by Student’s t-test.

### Data availability

All relevant data are available from the authors.

## Results and discussion

### Preparation and characterization of PtMn nanoparticles

**Fig. 1:**
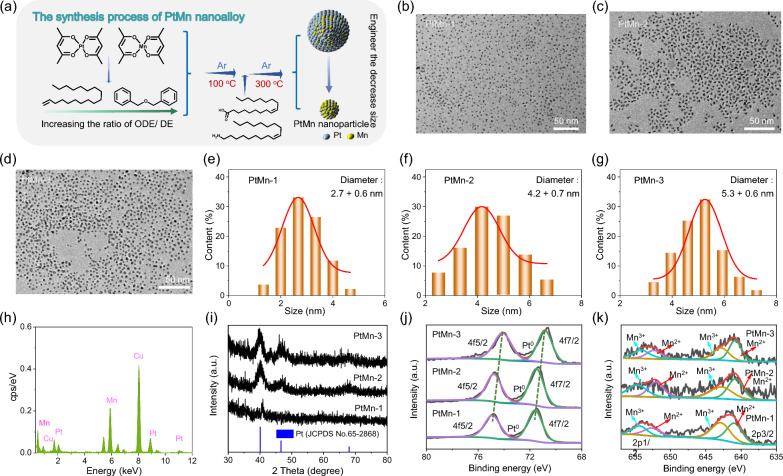
Synthesis and characterization of PtMn alloy nanoparticles. **a** Schematic illustration of the synthetic process for PtMn. **b** TEM image of PtMn-1 nanoparticles. **c** TEM image of PtMn-2 nanoparticles. **d** TEM image of PtMn-3 nanoparticles. **e**–**g** Size distribution histogram of over 100 randomly selected nanoparticles from the TEM images. **e** PtMn-1 nanoparticles from (**b**). **f** PtMn-2 nanoparticles from (**c**). **g** from PtMn-3 nanoparticles (**d**). **h** EDS spectra of PtMn-1. **i** Power XRD patterns of PtMn-1, PtMn-2 or PtMn-3. **j**, **k** XPS spectra of Pt 4f for PtMn-1, PtMn-2 or PtMn-3. **h** XPS spectra of Mn 2p for PtMn-1, PtMn-2 or PtMn-3

Firstly, a series of PtMn nanoparticles were synthesized by thermal decomposition, employing Mn(acac)_2_ as Mn precursor, Pt(acac)_2_ as Pt precursor, the mixed solution of oleic acid/ oleylamine as the reductant, and the mixture of 1-octadecene (ODE)/ dibenzyl ether (DE) as solvent agents, respectively (Fig. 1a). As shown in the transmission electron microscope (TEM) images, PtMn nanoalloy (PtMn-1, PtMn-2, PtMn-3) exhibited relatively uniform-sized nanoparticles with different diameters (2.7 ± 0.6, 4.2 ± 0.7 and 5.3 ± 0.6 nm, respectively) by adjusting the volume ratio of the solvent agents (ODE:DE = 1: 0; 1:1; 1:0, respectively) (Fig. 1b–g). Energy dispersive X-ray spectroscopy (EDS) illustrated that Pt and Mn elements existed on the PtMn-1 alloy (Fig. 1h). From powder X-ray diffraction pattern (XRD) of as-synthesized three kinds of PtMn nanoparticles, it was noted that PtMn-2 or PtMn-3 nanoparticles kept well consistent with standard Pt (JCPDS No.65-2868) crystalline structures, meanwhile the diffraction peaks of PtMn-1 broadened and the intensity decreased, which may be attributed to ultrasmall crystals (Fig. 1i) [55, 56]. X-ray photoelectron spectrum (XPS) of Pt 4f displayed binding energy peaks at 71 ± 0.3 eV and 74.5 ± 0.4 eV, which belonged to Pt^0^, demonstrating metallic Pt as substrate in three kinds of PtMn material (Fig. 1j and Additional file 1: Table S1). Especially, the binding energy peak of ultrasmall Pt nanoparticles (71.6 eV and 74.9 eV,) exhibited higher than larger Pt (71 and 74.1 eV; 71.4 and 74.7 eV), due to the initial and final state effects arising from their ultrasmall spatial extent [53, 55]. The binding energy peaks (654.7 ± 0.3 eV and 642.8 ± 0.1 eV; 652.3 ± 0.3 eV and 640.9 ± 0.1 eV) in XPS spectra of Mn 2p were ascribed to multivalent Mn (Mn^3+^ or Mn^2+^), respectively (Fig. 1k and Additional file 1: Table S2), confirming that PtMn nanoparticles contained Pt^0^ as the skeleton and multivalent Mn as the dopant [53].

Therefore, these data demonstrated the successful synthesis of a series of high-purity PtMn nanoparticles from 5.3 to 2.7 nm by adjusting the volume ratio of ODE/DE, which produced controllable size in a small range (Fig. 1a). The monodisperse nanocrystals with smaller dimensions probably enable the higher sensitivity in various bio applications, attributing from the increased surface-to-volume ratio with the size decrease [57, 58].

### pH-ultrasensitive Mn release, MRI imaging, and catalysis enhancement

**Fig. 2:**
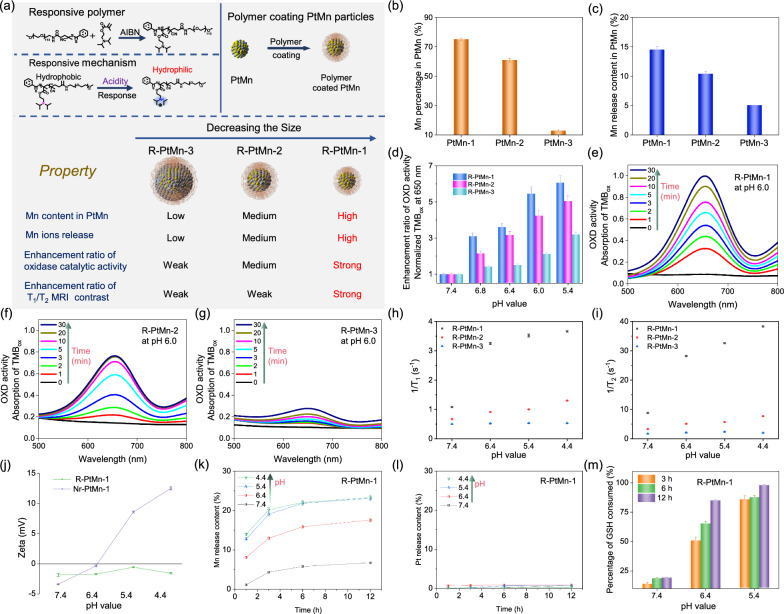
To compare pH-responsive polymer-coated PtMn-1, PtMn-2, or PtMn-3 (R-PtMn-1, R-PtMn-2, or R-PtMn-3) for best catalytic activity and MRI ability. **a** Schematic illustration for the synthetic process and property comparison of R-PtMn-1, R-PtMn-2, and R-PtMn-3. **b** The percentage of Mn content in three PtMn particles (n = 3). **c** The release percentage of Mn ions from uncoated PtMn-1, PtMn-2, or PtMn-3 particles (n = 3). **d** Enhancement ratio of oxidase catalytic activity: the absorption of TMB_ox_ at 650 nm (pH X/pH 7.4), calculated from Additional file 1: Fig. S6 (n = 3). Absorption spectra of TMB_ox_ incubated with R-PtMn-1 (**e**), R-PtMn-2 (**f**), or R-PtMn-3 (**g**) in HEPES buffer (pH = 6.0) at different incubation times. **h** 1/T_1_ or **i** 1/T_2_ of R-PtMn-1, R-PtMn-2, and R-PtMn-3 at the same total concentration in HEPES buffer with different pH values (n = 3). **j** Zeta potential for Nr-PtMn-1 or R-PtMn-1 in different pH values (n = 3). **k**, **l** Release profile of metal ions from R-PtMn-1 incubated in different pH values. **k** Mn or **l** Pt ions (n = 3). **m** The percentage of GSH-consumed for R-PtMn-1 in different pH conditions for different incubation times (n = 3)

The Pt or Mn content in three PtMn nanoalloys was measured via inductively coupled plasma–mass spectrometry (ICP-MS) and thus the chemical formulas of PtMn-1, PtMn-2, or PtMn-3 should be Pt_3_Mn_34_, PtMn_6_ or Pt_2_Mn, respectively, as shown in Additional file 1: Table S3. Additionally, we found that the more Mn content doping in ultrasmall PtMn-1 compared with the larger-sized PtMn-2 or PtMn-3 alloy (Fig. 2b). Next, we tested Mn release profile from three kinds of bare PtMn nanoparticles (without surface coating), via tetrahydrofuran solution containing PtMn nanoparticles incubation in the HEPES buffer (pH = 6.4). We found that higher content of Mn ions was released from PtMn-1 nanoparticles, compared with the larger-sized PtMn-2 or PtMn-3 alloy (Fig. 2c). We incubated PtMn-1 nanoparticles in acidic conditions (pH = 5.4) for 1 h and then conducted XPS characterization of them. From the XPS of Mn 2p, we found less Mn^2+^ and more Mn^3+^ resided in PtMn-1 nanoparticles after acidic treatment via quantifying the content of Mn^2+^/ Mn^3+^ in the alloy (Additional file 1: Fig. S1). Compared with PtMn-1 nanoparticles with no treatment of acid, we found less Mn^2+^ and more Mn^3+^ resided in PtMn-1 nanoparticles after acidic treatment. Thus, more Mn^2+^ ions were released from PtMn-1 nanoparticles triggered by acidity.

To realize the controlled Mn release from PtMn nanoparticles, pH-responsive amphiphilic polymer, and non-responsive polymer were synthesized, and tested by NMR spectra, confirming the successful synthesis (Additional file 1: Figs. S2 and S3) [53]. The pH-responsive amphiphilic polymer was tested by Matrix Assisted Laser Desorption Ionization Time of Flight Mass Spectrometry (MALDI-TOF) and Gel Permeation Chromatography (GPC), further confirming the successful synthesis (Additional file 1: Figs. S4 and S5). Additional file 1: Fig. S4 now presents the MALDI-TOF data, which confirms the successful synthesis of the polymers. The measured molecular weight of the polymers was found to be approximately 10,000–12000, which is greater than that of PEG (approximately 5000), further supporting the successful synthesis of the polymers. Additionally, Fig. S5 in the supplementary information provides the GPC data for the polymer. The results show a molecular weight of M_w_ = 1,043,581 g/mol, M_n_ = 4,072,813 g/mol, and a polymer dispersity index (PDI) of 3.9. The high M_w_ and M_n_ values observed further indicate the successful synthesis of the polymers. We appreciate your feedback, and these characterizations have significantly strengthened our manuscript. The revised version now includes the descriptions and figures in the Results and Discussion section, as well as the supporting information. Next, acidity-responsive polymer modified PtMn-1, PtMn-2, or PtMn-3 nanoparticles (R-PtMn-1, R-PtMn-2 or R-PtMn-3) or non- responsive polymer modified PtMn-1 nanoparticles (Nr-PtMn-1) were obtained, employing a nanoprecipitation process in Fig. 2a.

Next, we used a 3,3ʹ,5,5ʹ-tetramethylbenzidine (TMB) assay to determine the oxidase-like catalytic (OXD) activity. Firstly, we studied the OXD activity of R-PtMn-1, R-PtMn-2, or R-PtMn-3 under various HEPES buffers (pH = 7.4, 6.8, 6.4, 6.0, 5.4) (Fig. 2d and Additional file 1: Fig. S6) [59]. We discovered that R-PtMn-1 displayed higher OXD activity in acidity, while negligible activity in neutral conditions, indicating an acidity-activated OXD activity. Importantly, R-PtMn-1 still exhibited good OXD activity at very weak acidity (pH 6.8). Moreover, R-PtMn-1 exerted faster and better efficacy of catalytic activity than that of R-PtMn-2 or R-PtMn-3, probably owing to the fast and high release of Mn ions from PtMn. After quantification, the enhancement ratio in OXD activity (pH 6.0/pH 7.4) of R-PtMn-1 was markedly as high as 6.7 folds (Fig. 2d). As compared, the enhancement ratio in OXD activity of R-PtMn-2 or R-PtMn-3 was only 3.7 or 2.1 folds. We also compared the OXD catalytic ability for these PtMn nanoparticles in acidic HEPES buffer (pH = 6.0) with different incubation times (Fig. 2e–g). As for PtMn nanoparticles, the TMB_ox_ absorption change was increased with the incubation time increased in pH 6.0. The TMB_ox_ absorption change of R-PtMn-1 was significantly higher and faster than that of R-PtMn-2 or R-PtMn-3 at the same concentration. After quantification, the enhancement ratio in OXD activity (30 min /0 min) of R-PtMn-1 was markedly increased to 8.2 folds. For comparison, R-PtMn-2 or R-PtMn-3 has elevated only 5.3 or 2.5 folds. (Fig. 2f, g). According to the accurate and pH-ultrasensitive release of Mn ions from R-PtMn-1, we investigated the OXD activity of non-responsive polymer-coated PtMn (Nr-PtMn-1) in various pH buffers (pH = 7.4, 6.8, 6.4, 6.0, 5.4). Conversely, Nr-PtMn-1 showed nearly inactivated OXD activity in different pH values, due to little Mn release from Nr-PtMn-1 in acidity (Additional file 1: Fig. S7). It was expected that R-PtMn-1 could turn on the OXD capability of R-PtMn-1 in tumors while keeping the silence of catalytic activity in normal tissues.

According to the mild acidic of the tumor microenvironment (pH 6.4), a pH-responsive polymer was coated on the ultrasmall PtMn particles for precise controlled Mn release. We further detected the longitudinal and transverse relaxation time (1/T_1_ and 1/T_2_) relaxation time for three PtMn nanoparticles at the same concentration via Bruker Minispec analyzer (60 MHz). R-PtMn-1 exhibited the highest both 1/T_1_ and 1/T_2_ among three kinds of PtMn nanoparticles at the same total concentration. In pH 6.4, the 1/T_1_ and 1/T_2_ value enhancement contrast ratio of R-PtMn-1 (3 or 3.2 folds) is more sensitive, compared with R-PtMn-2 (1.4 or 1.5 folds) or R-PtMn-3 (1 or 1.2 folds) (Fig. 2h, i). As a result, R-PtMn-1 was chosen for further studies, owing to the most obvious enhancement ratio of catalytic activity and MRI contrast among those samples.

The hydrodynamic diameter of R-PtMn-1 showed no significant change in different solutions at different times via dynamic light scattering, meaning good compatibility and colloidal stability (Additional file 1: Fig. S8). Additionally, DLS sizes of R-PtMn-1, R-PtMn-2, and R-PtMn-3 nanoparticles exhibited no significant change, which indicated that the performance of different PtMn nanoparticles in regardless of DLS size (Additional file 1: Figs. S8 and S9). As the pH value changed from neutral condition (pH 7.4) to acidic condition (pH 4.4), R-PtMn-1 exhibited a continuous increase in zeta potential from − 3.4 to + 12.5 mV while Nr-PtMn-1 kept unchanged (Fig. 2j). Notably, DLS sizes of R-PtMn-1 exhibited a significant increase after incubation in various buffers for 24 h and found that the average DLS sizes of R-PtMn-1 were gradually reduced from ~ 50 nm to ~ 30 nm, which indicated that PtMn-1 were released as pH values decreasing from 7.4 to 5.4 (Additional file 1: Fig. S10). Furthermore, the metal ions released from R-PtMn-1 nanoparticles were analyzed via incubating in HEPES buffers with various pH, respectively by ICP-MS test (Fig. 2k and l). No measurable release of Mn ions from R-PtMn-1 at pH 7.4 was observed, whereas R-PtMn quickly released an amount of Mn ions in acidity (pH 5.4) (Fig. 2k). Furthermore, R-PtMn-1 could release more Mn ions as pH value decreasing from 7.4 to 4.4, testifying pH-triggered Mn release. Because of inert Pt^0^, we detected no obvious leakage of Pt ions released from R-PtMn-1 in various pH conditions, proving that ultrasmall alloy (< 3 nm) enabled it to use as an excellent Mn reservoir for high storage and sensitive release of Mn (Fig. 2l).

Owing to acidity-triggered Mn release, we measured the oxidization ability of glutathione (GSH). We measured the residual GSH content via colorimetric 5,5′-dithiobis-2-(nitrobenzoicacid) (DTNB) after incubation with R-PtMn-1 or Nr-PtMn-1 (Fig. 2m and Additional file 1: Fig. S11). At pH 7.4, little GSH was oxidized by R-PtMn-1, due to little leakage of Mn ions from R-PtMn-1. As the pH value dropped the time went on, and R-PtMn-1 released much more Mn ions and consumed more GSH content. More than 80% of GSH content was consumed by R-PtMn-1 at pH 5.4 within 3 h incubation because a large amount of released Mn ions was triggered by the acidity and further oxidized GSH. As a control, less than 20% of the GSH level was oxidized by Nr-PtMn-1, attributing to blocking Mn ions release via the inert polymer.

Thus, the catalytic activity and magnetic properties of nanoparticles are very sensitive to the decrease in the size of PtMn alloy. As the core size varied from PtMn-3 (5.3 nm) to PtMn-1 (2.7 nm), the enhancement ratio in oxidase activity (2.1 folds), 1/T_1_ value (1.1 folds) or 1/T_2_ value (1.2 folds), gradually increased to 6.7 folds, 3 folds or 3.2 folds, respectively, (Fig. 2a). When in pH 7.4, R-PtMn-1 showed no measurable oxidase catalytic activity and relatively low MRI values, improving accurate cancer theranostic from normal tissue.

### High-specificity cancer therapy efficacy in vitro

**Fig. 3:**
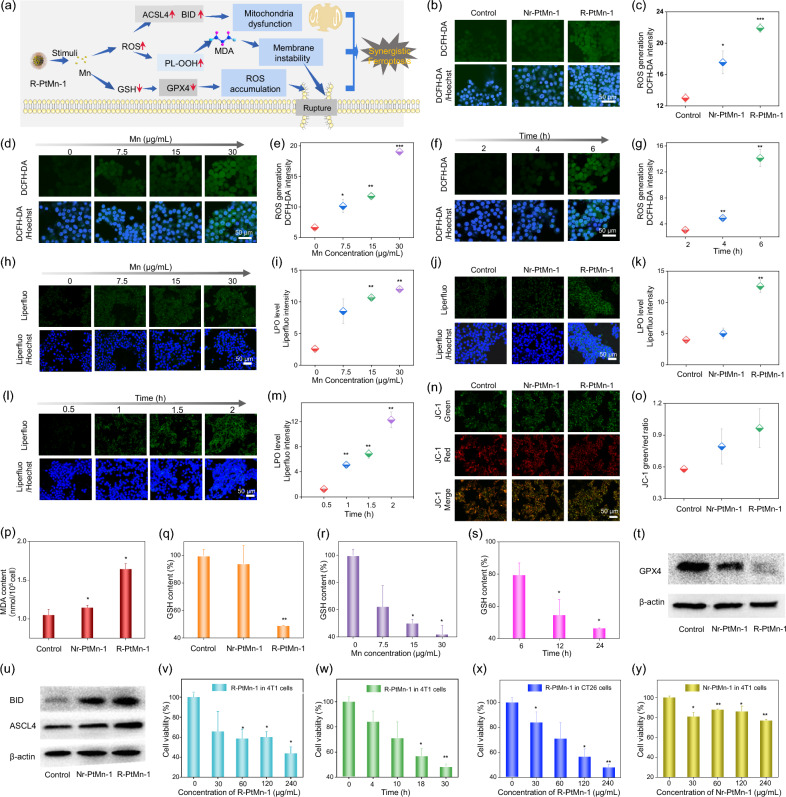
High-specificity cancer therapy efficacy in vitro. **a** Schematic illustration for R-PtMn-1 involved in the ferroptosis process. Confocal images (**b**) and corresponding fluorescent intensity (**c**) of DCHF-DA-stained 4T1 cells pre-treated Nr-PtMn-1 or R-PtMn-1. Confocal images (**d**) and corresponding fluorescent intensity (**e**) of DCHF-DA-stained 4T1 cells pre-treated with R-PtMn-1 with different Mn concentrations. Confocal images (**f**) and corresponding fluorescent intensity (**g**) of DCHF-DA-stained 4T1 cells pre-treated with R-PtMn-1 for different incubation times. Confocal images (**h**) and corresponding fluorescent intensity (**i**) of liperfluo-stained 4T1 cells pre-treated with R-PtMn-1 with different Mn concentrations. Confocal images (**j**) and corresponding fluorescent intensity (**k**) of liperfluo-stained 4T1 cells pre-treated with Nr-PtMn-1 or R-PtMn-1. Confocal images (**l**) and corresponding fluorescent intensity (**m**) of liperfluo-stained 4T1 cells pre-treated with R-PtMn-1 for different incubation times. Confocal images (**n**) and the ratio of green/ red fluorescent intensity (**o**) of JC-1 stained 4T1 cells pretreated with Nr-PtMn-1 or R-PtMn-1. **p** Intracellular MDA level for 4T1 cells treated with Nr-PtMn-1 or R-PtMn-1. **q** Intracellular GSH level for 4T1 cells treated with Nr-PtMn-1 or R-PtMn-1. **r** Intracellular GSH level for 4T1 cells treated with R-PtMn-1 with different Mn concentrations. **s** Intracellular GSH level for 4T1 cells treated with R-PtMn-1 for different incubation times. **t**–**u** The expression of Western blotting assay for 4T1 cells treated with Nr-PtMn-1 or R-PtMn-1. **t** GPX4 protein. **u** ACSL-4 and BID protein. **v** The relative cellular viability of 4T1 cells treated with various concentrations of R-PtMn-1 for 24 h. **w** The relative cellular viability of 4T1 cells treated with R-PtMn-1 (240 μg/mL) at different times. **x** The relative cellular viability of CT26 cells treated with various concentrations of R-PtMn-1 for 24 h. **y** The relative cellular viability of 4T1 cells treated with various concentrations of Nr-PtMn-1 for 24 h. The statistical analysis was performed in contrast to the control group (*p < 0.05, **p < 0.01, ***p < 0.001, t-test)

To establish the pH ultrasensitive activation which could “turn on” the efficient OXD catalytic capability, we evaluated intracellular ROS production, lipid peroxidation level, mitochondrial membrane potential change, malondialdehyde (MDA) content, GSH level and western blotting assay for R-PtMn-1, while using inert particles (Nr-PtMn-1) as the control group (Fig. 3a). As shown in confocal images of tumor cells stained with 2′,7′-dichlorofluorescin diacetate (DCFH-DA) as a ROS indicator (Fig. 3b, c), strong green fluorescence was observable in those cells treated with R-PtMn-1, while weak fluorescence for Nr-PtMn-1, proving that more ROS generation compared with Nr-PtMn-1. As Mn concentration or the incubation time increased, we found that the green fluorescence intensity from those cells treated with R-PtMn-1 was gradually improved (Fig. 3d–g). As exhibited in confocal images of liperfluo (LPO indicator) staining cancer cells and their quantification, R-PtMn-1 induced higher LPO levels than Nr-PtMn-1 (Fig. 3j, k). As shown in Fig. 3h, i and l, m, the green fluorescence intensity for those cells treated with R-PtMn-1 was gradually enhanced, by increasing Mn concentration or incubation time. Encouraged by the damage caused by intracellular lipid peroxidation to organelles, the 5,5ʹ,6,6ʹ-tetrachloro-1,1ʹ,3,3ʹ-tetraethylimidacarbocyanine iodide (JC-1) staining was used to measure the mitochondrial membrane potential change (Fig. 3n, o). As depicted in JC-1 staining cells images, R-PtMn-1-treated cancer cells showed a higher green/red ratio, compared with Nr-PtMn-1 treated cancer cells, meaning severe mitochondrial disorder for enhanced ferroptosis. According to malondialdehyde (MDA) as an end-product of LPO, the intracellular MDA content was tested via MDA assay and found higher yield for the R-PtMn-1 group, in contrast with the Nr-PtMn-1 (Fig. 3p). Moreover, the intracellular GSH level was detected by DTNB assay kit, after cancer cells incubation with R-PtMn-1 or Nr-PtMn-1. The content of GSH in R-PtMn-1 was greatly reduced, compared with that of Nr-PtMn-1 (Fig. 3q), attributed to effective GSH consumption. As Mn concentration increased, much more overproduced GSH would be effectively depleted (Fig. 3r). As the incubation time went on, the GSH content would continuously decrease (Fig. 3s). Mn ions were released for generating ROS or oxidative stress damage via Fenton-like, Haber–Weiss or oxidation reactions. Meanwhile, Mn ions could be reduced and consume the reducing substances within the tumor (e.g., GSH). These reasons thereby induce the inactivation of glutathione-dependent peroxidases 4 (GPX4). Subsequently, we detected the expression of glutathione peroxidase 4 (GPX4), acyl-CoA synthetase long-chain family member (ACSL4), and recombinant BH3 interacting domain death agonist (BID) protein via western blotting assay. R-PtMn-1-treated cancer cells caused GPX4 protein downregulation, compared with that incubated with the Nr-PtMn-1 group (Fig. 3t and Additional file 1: Fig. S12a). The involvement of Mn in electron transfer reactions, activation of molecular oxygen, and other biological processes highlights its importance as an essential micronutrient [1]. Additionally, the intrinsic activities of GPx- and CAT-like activities exhibited by Mn-nanomaterials, attributed to the variable Mn valent states and morphology, further support the downregulation of GPX4 upon Mn release [16–18]. The release of Mn ions can generate reactive oxygen species (ROS) or oxidative stress damage through Fenton-like, Haber–Weiss, or oxidation reactions. [12, 53] Moreover, Mn ions can be reduced and consume reducing substances within the tumor, such as glutathione (GSH) [53]. These reasons provide a plausible explanation for the inactivation of GPX4. R-PtMn-1-treated cancer cells induced the up-regulation of BID or ASCL4 protein, compared with Nr-PtMn-1-treated cells, probably due to the oxidase-like catalytic capability (Fig. 3u and Additional file 1: Fig. S12b, c).

Methyl thiazolyl tetrazolium (MTT) assay was performed to test the relative intracellular viability of R-PtMn-1 or Nr-PtMn-1 against cancer cells [mouse murine breast cancer cells (4T1) or mouse colon cancer cells (CT26)] (Fig. 3v–y). R-PtMn-1 effectively inhibited the cellular viability of cancer cells (4T1 or CT26) and the long incubation time induced better cancer inhibition. As controlled, we found that no significant cytotoxicity was observed after the 4T1 cells were incubated with Nr-PtMn-1 at different concentrations (Fig. 3y). In addition, we measured the ability of intracellular anticancer activity (4T1 or CT26) for those three kinds of nanoparticles (Additional file 1: Figs. S13, 14). We discovered that R-PtMn-1 possessed the highest anticancer activity, attributed to the good OXD activity in the solution.

To investigate its safety effects on normal cells, we incubated R-PtMn-1 with normal cells (HEK293). From DCFH-DA or liperfluo staining confocal imaging, our findings revealed no noticeable intracellular ROS or LPO in R-PtMn-1-treated HEK293 cells, suggesting that R-PtMn-1 does not induce oxidative injury in normal cells (Additional file 1: Figs. S15 and S16). The impact of R-PtMn-1 on the cellular viability of normal cells (HEK293) was also investigated. No notable cytotoxicity was observed with R-PtMn-1 treatment (Additional file 1: Fig. S17). These results demonstrated that R-PtMn-1 induced no endogenous injury toward normal cells.

In the tumor microenvironment, much more released Mn^2+^/Mn^3+^ could improve the oxidase catalytic efficiency, promoting BID and ASCL4 activation. Meanwhile, Mn^3+^/Mn^4+^ could consume overproduced GSH within the tumor, favoring GPX4 inactivation. The down-regulation of GPX4 can facilitate the rate of free radical chain reaction and the up-regulation of ACSL4 results in incorporating more polyunsaturated fatty acids into cellular phospholipids (especially phosphatidylethanolamine), boosting the lipid peroxidation level for amplified ferroptosis (Fig. 3a). Hence, the acidity-activated R-PtMn-1 could efficiently provoke ferroptosis mediated cancer therapy via controlled Mn accumulation within tumor cells, GSH consumption, lipid peroxidation elevation, and mitochondrial disorder, as well as down-regulation of GPX4 and up-regulation of ACSL4 and BID.

### pH ultrasensitive high-field T_1_-T_2_ dual model MRI contrast

**Fig. 4:**
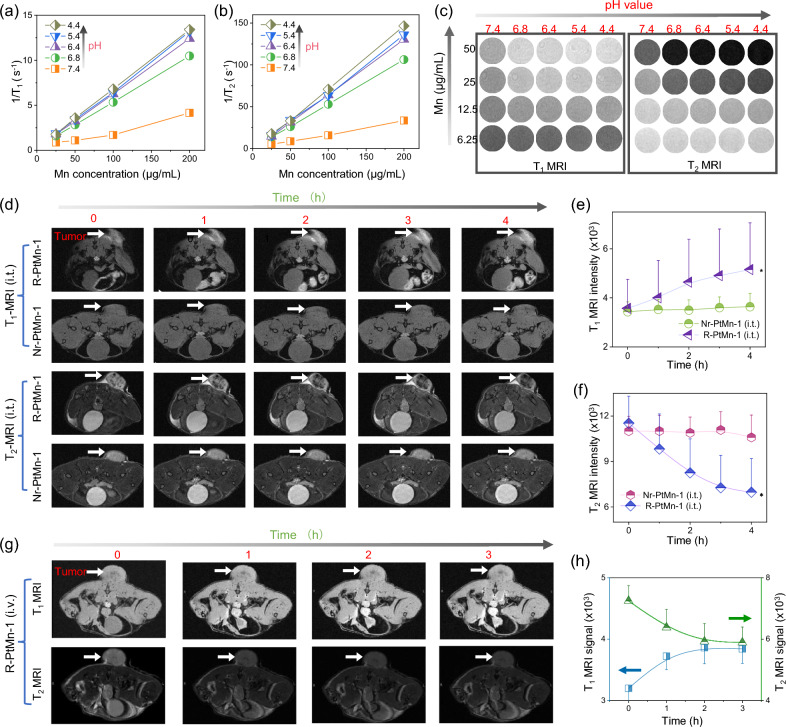
pH ultrasensitive high-field T_1_–T_2_ dual model MRI contrast. Plotting 1/T_1_ (**a**) or 1/T_2_ (**b**) versus different concentrations of R-PtMn-1 in HEPES buffer with different pH conditions (2 ×, pH = 7.4, 6.8, 6.4, 5.4, 4.4). **c** T_1_- or T_2_-MRI (7 T) images for different concentrations of R-PtMn-1 in HEPES buffer with different pH conditions (2 ×, pH = 7.4, 6.8, 6.4, 5.4, 4.4). **d** T_1_-MRI or T_2_-MRI images for 4T1 tumor bearing mice at various times, after the local injection of Nr-PtMn-1 or R-PtMn-1 into the tumor. **e**–**f** Quantification of T_1_-MRI or T_2_-MRI signal intensity of tumor areas from (**d**). **g** T_1_-MRI or T_2_-MRI images of mice at various times, after *i.v.* injection of R-PtMn-1. **h** Quantification of T_1_-MRI or T_2_-MRI signal intensity of tumor areas from (**g**). The statistical analysis was performed in contrast to the control group (*p < 0.05, t-test)

Triggered by very mild acidity (pH 6.8), R-PtMn-1 could obtain the enhancement of MRI contrast through Bruker Minispec analyzer (60 MHz) and high-field MRI scanner (7 T), respectively (Fig. 4a–c). As the pH value decreased, the r_1_ or r_2_ value of R-PtMn-1 was distinctly enhanced, suggesting acidity-activated MRI contrast ability for R-PtMn-1 (Fig. 4a, b). As depicted in the MRI signal of R-PtMn-1 in buffer (Fig. 4c), R-PtMn-1 presented a pH ultrasensitive T_1_ or T_2_-MRI contrast enhancement. As the pH value decreased, T_1_-weighted MRI images exhibited great brightness and T_2_-weighted MRI images exhibited great darkness. Especially in pH 6.8, PtMn still displayed an obvious change in T_1_-/T_2_- high field MRI images, compared with that in neutral.

Whereafter, the in vivo pH-responsive MRI enhancement of R-PtMn-1 was investigated, via local injection of R-PtMn-1 into the tumor, meanwhile employing pH-insensitive Nr-PtMn-1 as control, using a 7 T scanner (Fig. 4d–f). From MRI images, T_1_-MRI of the tumor area became positive enhancement, meanwhile, T_2_-MRI became negative enhancement darkened gradually from 0 to 4 h, demonstrating that the acidity in the tumor would enable to amplify T_1_- or T_2_-MRI contrast. As a control, the area of the tumor injected with Nr-PtMn-1 exhibited no significant fluctuation of the contrast and intensity from T_1_- or T_2_-MRI images at different times. To detect the tumor-targeted performance, the mouse was intravenous injected with R-PtMn-1, and then scanned (Fig. 4g, h). The tumor areas of the mouse showed a positive enhancement in T_1_-MRI images and a negative growth in T_2_-MRI images, which was the intravenous injection of R-PtMn-1 for 3 h, proving the efficient enrichment of the tumor.

Meaningfully, PtMn-1 nanoalloy, harbored antiparallel magnetic moments, can be refractory to the external magnetic field to achieve T_1_/T_2_ high-field MRI contrast [60]. Even in pH 6.8, R-PtMn-1 could exhibit a significant enhancement of MRI contrast in solution. It is very noteworthy that the tumor areas (pH 6.4–6.8) could show considerable contrast enhancement in high field T_1_- or T_2_-MRI, demonstrating that ultrasensitive pH-responsive. Thus, R-PtMn-1 possess extraordinary prospect for further biological MRI applications.

### Tumor-specific cancer therapy in vivo

**Fig. 5:**
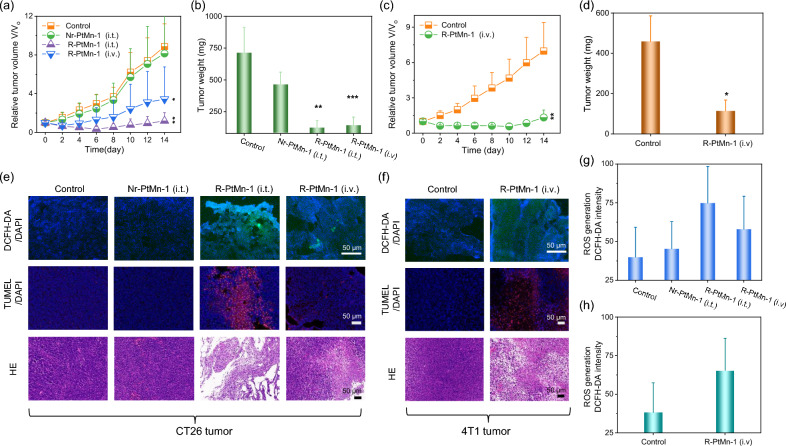
Tumor-specific cancer therapy in vivo. **a**, **b** CT26 tumors bearing mice were divided into four groups (n = 5) and received the following administration (1) None treatment as the control group; (2) Nr-PtMn-1 (*i.t.*); (3) R-PtMn-1 (*i.t.*); (4) R-PtMn-1 (*i.v.*). **a** Tumor growth curves of different groups. **b** Tumor weight of each group on the 14th day post-treatment. **c**, **d** 4T1 tumors bearing mice were divided into two groups (n = 5) and received the following administration (1) None treatment as the control group; (2) R-PtMn-1 (*i.v.*). **c** Tumor growth curves of different groups. **d** Tumor weight of each group on the 14th day post-treatment. **e**–**h** Nr-PtMn-1 or R-PtMn-1 was locally injected into the tumor, followed by tissue staining via DCFH-DA, or TUNEL, respectively. The representative tumors were collected from the mice in each group for various stainings, such as DCFH-DA staining for ROS generation; and TUNEL and H&E staining for apoptosis and necrosis. **e** Representative confocal images of DCFH-DA CT26 tumor slice, as well as TUNEL or H&E-stained tumor slice. **f** Representative confocal images of DCFH-DA or liperfluo-stained 4T1 tumor slice, as well as TUNEL or H&E-stained tumor slice. **g** Quantification of fluorescence intensity for DCFH-DA from (**e**). **h** Quantification of fluorescence for DCFH-DA from (**f**). The statistical analysis was performed in contrast to the control group (*p < 0.05, **p < 0.01, ***p < 0.001, t-test)

We further explored the anticancer effect of R-PtMn-1 in vivo. mouse colon cancer cells (CT26) tumors bearing female mice were randomly divided into 4 groups and followed the following treatments (1) None treatment as control group; (2) Nr-PtMn-1 (25 µL, Mn: 0.7 mg/mL, *i.t.*); (3) R-PtMn-1 (25 µL, Mn: 0.7 mg/mL, *i.t.*); (4) R-PtMn-1 (200 µL, Mn: 0.7 mg/mL, *i.v.*). It is noted that the CT26 tumor growth and tumor weights were considerably suppressed in those mice *i.t.* or *i.v.* injected with R-PtMn-1, in contrast to Nr-PtMn-1 or the control group, exhibiting a great anticancer activity (Fig. 5a, b). Furthermore, we proceeded to evaluate the tumor growth inhibitory performance of R-PtMn-1 via murine breast cancer cells (4T1) tumors bearing female mice were divided into 2 groups and then followed the injection (1) None treatment as control group; (2) R-PtMn-1 (200 µL, Mn: 0.7 mg/mL, *i.v.*), R-PtMn-1 was able to suppress the 4T1 tumor growth notably through the change in growth curves and weights of the tumor (Fig. 5c, d).

The therapeutic efficacies of various treatments were further evaluated via various staining with CT26 or 4T1 tumor sections (Fig. 5e–j). As exhibited in DCFH-DA-stained tumor images and their quantifications (Fig. 5e, f), both *i.t.* and *i.v.* treatment of R-PtMn-1 led to higher oxidative stress toward CT26 or 4T1 tumor than in Nr-PtMn-1 or control group (Fig. 5g, h). In addition, hematoxylin and eosin (HE) and terminal deoxynucleotidyl transferase–mediated deoxyuridine triphosphate nick end labeling (TUNEL) staining images of tumor slices further defined serious damages induced by R-PtMn-1, while negligible injure for Nr-PtMn-1 or control groups (Fig. 5e, f). These results demonstrated that R-PtMn-1 exhibited good tumor-specific ferroptosis therapeutically. There were no appreciable fluctuations in mice weights of each group, which were recorded during 2 weeks of therapy by recording mice’s body weight every two days. H&E-stained major organs (heart, liver, spleen, lung, and kidney) from each group were collected after 14 days and displayed no significant toxicities and inflammatory lesions (Additional file 1: Figs. S18–21*)*, demonstrating high biocompatibility for safe in vivo therapy.

In consequence, the extracellular pH within the tumor microenvironment is weak acidity (pH 6.4–6.8), which is lower than that of normal tissue or bloodstream (pH 7.2–7.4). The pH difference between normal tissues and tumors has been extensively utilized as a trigger for ultra pH-responsive delivery of R-PtMn-1. When circulated in blood or normal tissues, OXD activity and MRI of R-PtMn-1 were both able to keep off, effectively avoiding the premature leakage and false signal during the delivery process ahead of reaching the tumor, improving precise theranostic and reducing the off-target toxicity toward normal tissues. When accumulated within the tumor, according to pH ultrasensitive oxidase enzyme-like activity, R-PtMn-1 was able to incur efficient ferroptosis within CT26 or 4T1 tumor.

## Conclusion

We have prepared a series of different-sized PtMn alloy nanoparticles from 2.7 to 5.3 nm and found that the enhancement ratio of OXD activity and MRI contrast would be markedly improved by the reduction of size. Furthermore, PtMn-1 nanoparticles (< 3 nm) are developed as a pH-ultrasensitive manganese-based agent, and even in mild acidity (pH 6.8) without the requirement of H_2_O_2_. Additionally, R-PtMn-1 as a sensitive manganese release modulator could also exert mildly acidity-triggered T_1_-/T_2_- MRI enhancement within the tumor, due to the highest enhancement contrast ratio among PtMn particles. Collectively, the ultrasensitive catalytic and MRI ability of the R-PtMn-1 nanoplatform may pave the way for future biomedical theranostics.

### Supplementary Information

**Additional file 1: Table S1.** The detailed peak positions for the fitted Pt peaks. **Table S2.** The detailed peak positions for the fitted Mn peaks. **Table S3.** The summary of added precursor ratios and obtained element percent for various PtMn nanoparticles was determined from ICP. **Fig. S1 **(a) XPS spectra of Mn 2p for PtMn-1 after incubation in HEPES (10x, 5.4) at room temperature for 1 h. (b) The percentage of Mn^2+^ or Mn^3+^ within PtMn-1 before and after incubation in acidic conditions (pH 5.4), determined from XPS in Figure 1k and Figure S1a. **Fig. S2 **^1^H NMR of non-responsive polymer. **Fig. S3 **^1^H NMR of pH-responsive polymer. **Fig. S4 **MALDI-TOF of pH-responsive polymer. **Fig. S5 **GPC tests of pH-responsive polymer. **Fig. S6 **Absorption spectra of TMB_ox_ incubated with R-PtMn-1 (**a**), R-PtMn-2 (**b**) or R-PtMn-3 (**c**) in HEPES buffer at different pH (pH= 7.4, 6.8, 6.4, 6.0, or 5.4). **Fig. S7 **Absorption spectra of TMB_ox_ incubated with Nr-PtMn-1 in different pH values. **Fig. S8 **Hydrodynamic size of R-PtMn-1 dispersed in H_2_O, DPBS, HEPES, or DMEM for different times. **Fig. S9 **Hydrodynamic size of R-PtMn-2 or R-PtMn-3 dispersed in H_2_O, DPBS, HEPES, or DMEM for 12 h. **Fig. S10** Hydrodynamic size of R-PtMn-1 dispersed in HEPES buffer with different pH values for 12 h. **Fig. S11 **The percentage of GSH-consumed for Nr-PtMn-1 in different pH at 12 h. **Fig. S12** (a) Quantitative analysis of relative GPX4 levels. (b) Quantitative analysis of relative BID levels. (c) Quantitative analysis of relative ASCL4 levels. **Fig. S13** The relative cellular viability of 4T1 cancer cells treated with various concentrations of R-PtMn-2 (**a**) or R-PtMn-3 (**b**) for 24 h. **Fig. S14 **The relative cellular viability of CT26 cancer cells treated with various concentrations of R-PtMn-2 (**a**) or R-PtMn-3 (**b**) for 24 h. **Fig. S15 **Confocal images of DCHF-DA-stained HEK293 normal cells pre-treated R-PtMn-1. **Fig. S16 **Confocal images of liperfluo-stained HEK293 normal cells pre-treated R-PtMn-1. **Fig. S17 **The relative cellular viability of HEK293 normal cells treated with various concentrations of R-PtMn-1 for 24 h. **Fig. S18 **Body weights of mice bearing CT26 tumor from each group post various treatments were recorded every other day during 14 days study. **Fig. S19 **Body weights of mice bearing 4T1 tumor from each group post various treatments were recorded every other day during 14 days’ of study.** Fig. S20 **H&E-stained images of major organs in CT26 tumors bearing mice from one of each group. Those organs were collected on the 14th day post various injections.** Fig. S21 **H&E-stained images of major organs in 4T1 tumors bearing mice from each group. Those organs were collected on the 14th day post various injections.

## Data Availability

The data used and/or analyzed to support the current study are available from the corresponding author upon reasonable request.
