# Supplementary material for: Ultrasmall PtMn nanoparticles as sensitive manganese release modulator for specificity cancer theranostics
Source: J Nanobiotechnology. 2023 Nov 18;21:434. doi: 10.1186/s12951-023-02172-y (PMC10657629; doi:10.1186/s12951-023-02172-y)
Supplement: Supplementary file 1 — Additional file 1: Table S1. The detailed peak positions for the fitted Pt peaks. Table S2. The detailed peak positions for the fitted Mn peaks. Table S3. The summary of added precursor ratios and obtained element percent for various PtMn nanoparticles was determined from ICP. Fig. S1 (a) XPS spectra of Mn 2p for PtMn-1 after incubation in HEPES (10x, 5.4) at room temperature for 1 h. (b) The percentage of Mn2+ or Mn3+ within PtMn-1 before and after incubation in acidic conditions (pH 5.4), determined from XPS in Figure 1k and Figure S1a. Fig. S2 1H NMR of non-responsive polymer. Fig. S3 1H NMR of pH-responsive polymer. Fig. S4 MALDI-TOF of pH-responsive polymer. Fig. S5 GPC tests of pH-responsive polymer. Fig. S6 Absorption spectra of TMBox incubated with R-PtMn-1 (a), R-PtMn-2 (b) or R-PtMn-3 (c) in HEPES buffer at different pH (pH= 7.4, 6.8, 6.4, 6.0, or 5.4). Fig. S7 Absorption spectra of TMBox incubated with Nr-PtMn-1 in different pH values. Fig. S8 Hydrodynamic size of R-PtMn-1 dispersed in H2O, DPBS, HEPES, or DMEM for different times. Fig. S9 Hydrodynamic size of R-PtMn-2 or R-PtMn-3 dispersed in H2O, DPBS, HEPES, or DMEM for 12 h. Fig. S10 Hydrodynamic size of R-PtMn-1 dispersed in HEPES buffer with different pH values for 12 h. Fig. S11 The percentage of GSH-consumed for Nr-PtMn-1 in different pH at 12 h. Fig. S12 (a) Quantitative analysis of relative GPX4 levels. (b) Quantitative analysis of relative BID levels. (c) Quantitative analysis of relative ASCL4 levels. Fig. S13 The relative cellular viability of 4T1 cancer cells treated with various concentrations of R-PtMn-2 (a) or R-PtMn-3 (b) for 24 h. Fig. S14 The relative cellular viability of CT26 cancer cells treated with various concentrations of R-PtMn-2 (a) or R-PtMn-3 (b) for 24 h. Fig. S15 Confocal images of DCHF-DA-stained HEK293 normal cells pre-treated R-PtMn-1. Fig. S16 Confocal images of liperfluo-stained HEK293 normal cells pre-treated R-PtMn-1. Fig. S17 The relative cellular viability of HEK2 [file 12951_2023_2172_MOESM1_ESM.docx]

**Additional file 1 for**

**Ultrasmall PtMn Nanoparticles as Sensitive Manganese Release Modulator for Specificity Cancer Theranostics**

Guoqiang Guan^1,2^, Huiyi Liu^2^, Juntao Xu^2^, Qingpeng Zhang^2^, Zhe Dong^2^, Lingling Lei^2^, Cheng Zhang^2^, Renye Yue^2^, Hongchang Gao^1,^*, Guosheng Song^2,^*, Xian Shen^1,^*

1. Department of Gastrointestinal Surgery, Key Laboratory of Diagnosis and Treatment of Severe Hepato-Pancreatic Diseases of Zhejiang Province, The First Affiliated Hospital of Wenzhou Medical University, Oujiang Laboratory, Wenzhou, Zhejiang Province, 325000, China.

2. State Key Laboratory for Chemo/ Bio-Sensing and Chemometrics, College of Chemistry and Chemical Engineering, Hunan University, Changsha 410082, China.

* Corresponding author: E-mail: [gaohc27@wmu.edu.cn](mailto:gaohc27@wmu.edu.cn) (Hongchang Gao); [songgs@hnu.edu.cn](mailto:songgs@hnu.edu.cn) (Guosheng Song); [shenxian@wmu.edu.cn](mailto:shenxian@wmu.edu.cn) (Xian Shen).

**Additionalas file**

| Sample | Pt 4f5/2 | Pt 4f7/2 |
| --- | --- | --- |
| PtMn-1 | 74.9 | 71.6 |
| PtMn-2 | 74.7 | 71.4 |
| PtMn-3 | 74.1 | 71 |

**Table S1.** The detailed peak positions for the fitted Pt peaks.

| Sample | Mn2p1/2 | Mn2p1/2 | Mn2p3/2 | Mn2p3/2 |
| --- | --- | --- | --- | --- |
| PtMn-1 | 654.4 | 652.6 | 642.9 | 641 |
| PtMn-2 | 654.7 | 652.1 | 642.8 | 640.8 |
| PtMn-3 | 654.1 | 652.3 | 642.8 | 640.8 |

**Table S2.** The detailed peak positions for the fitted Mn peaks.

|  | Precursor for  synthesis | Final products | | |
| --- | --- | --- | --- | --- |
| Sample | Molar ratio  (ODE: DE) | Mass ratio  (Pt: Mn) | Molar ratio  (Pt: Mn) | chemical formula |
| PtMn-1 | 1:0 | 23.9：76.1 | 8.1：91.9 | Pt_3_Mn_34_ |
| PtMn-2 | 1:1 | 37.2：62.9 | 14.1：85.9 | PtMn_6_ |
| PtMn-3 | 0:1 | 87.6：12.4 | 66.6：33.4 | Pt_2_Mn |

**Table S3.** The summary of added precursor ratios and obtained element percent for various PtMn nanoparticles was determined from ICP.

**Fig. S1** (a) XPS spectra of Mn 2p for PtMn-1 after incubation in HEPES (10x, 5.4) at room temperature for 1 h. (b) The percentage of Mn^2+^ or Mn^3+^ within PtMn-1 before and after incubation in acidic conditions (pH 5.4), determined from XPS in Figure 1k and Figure S1a.

**Fig. S2** ^1^H NMR of non-responsive polymer.

**Fig. S3** ^1^H NMR of pH-responsive polymer.

**Fig. S4** MALDI-TOF of pH-responsive polymer.

**Fig. S5** GPC tests of pH-responsive polymer.

**Fig. S6** Absorption spectra of TMB_ox_ incubated with R-PtMn-1 **(a)**, R-PtMn-2 **(b)** or R-PtMn-3 **(c)** in HEPES buffer at different pH (pH= 7.4, 6.8, 6.4, 6.0, or 5.4).

**Fig. S7** Absorption spectra of TMB_ox_ incubated with Nr-PtMn-1 in different pH values.

**Fig. S8** Hydrodynamic size of R-PtMn-1 dispersed in H_2_O, DPBS, HEPES, or DMEM for different times.

**Fig. S9** Hydrodynamic size of R-PtMn-2 or R-PtMn-3 dispersed in H_2_O, DPBS, HEPES, or DMEM for 12 h.

**Fig. S10** Hydrodynamic size of R-PtMn-1 dispersed in HEPES buffer with different pH values for 12 h.

**Fig. S11** The percentage of GSH-consumed for Nr-PtMn-1 in different pH at 12 h.

**Fig. S12** (a) Quantitative analysis of relative GPX4 levels. (b) Quantitative analysis of relative BID levels. (c) Quantitative analysis of relative ASCL4 levels.

**Fig. S13** The relative cellular viability of 4T1 cancer cells treated with various concentrations of R-PtMn-2 **(a)** or R-PtMn-3 **(b)** for 24 h.

**Fig. S14** The relative cellular viability of CT26 cancer cells treated with various concentrations of R-PtMn-2 **(a)** or R-PtMn-3 **(b)** for 24 h.

**Fig. S15** Confocal images of DCHF-DA-stained HEK293 normal cells pre-treated R-PtMn-1.

**Fig. S16** Confocal images of liperfluo-stained HEK293 normal cells pre-treated R-PtMn-1.

**Fig. S17** The relative cellular viability of HEK293 normal cells treated with various concentrations of R-PtMn-1 for 24 h.

**Fig. S18** Body weights of mice bearing CT26 tumor from each group post various treatments were recorded every other day during 14 days study.

**Fig. S19** Body weights of mice bearing 4T1 tumor from each group post various treatments were recorded every other day during 14 days’ of study.

**Fig. S20** H&E-stained images of major organs in CT26 tumors bearing mice from one of each group. Those organs were collected on the 14^th^ day post various injections.

**Fig. S21** H&E-stained images of major organs in 4T1 tumors bearing mice from each group. Those organs were collected on the 14^th^ day post various injections.
